# Patient's Death From the Perspective of Nursing Students

**DOI:** 10.3389/fpubh.2021.636582

**Published:** 2021-05-14

**Authors:** Magdalena Szczupakowska, Patrycja Stolarek, Magdalena Roszak, Katarzyna Głodowska, Ewa Baum

**Affiliations:** ^1^Department of Social Sciences and the Humanities, Faculty of Health Sciences, Poznan University of Medical Sciences, Poznan, Poland; ^2^Department of Computer Science and Statistics, Poznan University of Medical Sciences, Poznan, Poland; ^3^Division of Philosophy of Medicine and Bioethics, Faculty of Health Sciences, Poznan University of Medical Sciences, Poznan, Poland; ^4^Division of Philosophy of Medicine and Bioethics, Department of Social Sciences and the Humanities, Faculty of Health Sciences, Poznan University of Medical Sciences, Poznan, Poland

**Keywords:** nursing students, education, death, student's perception, undergraduate, patient's death

## Abstract

**Introduction:** An exceedingly small amount of scientific research concerns the response to patient death among nursing students. There was a need to examine their perspective on patient death with which they experience during their studies. The authors wanted to check the subjective assessment of students' preparation for patient death and their perception of classes conducted in this area.

**Methods:** The research used the diagnostic survey method and was conducted in May 2019 using Google Form on social media. The 467 nursing students answered 14 questions in the original questionnaire about their experience with patient death. The analyzed data were expressed as median, minimum and maximum values, or percentage, as appropriate. Comparison of groups was performed using the Mann–Whitney *U*-test or the Kruskal–Wallis test. The relationship between variables was analyzed with Spearman correlation coefficient or contingency coefficient (the χ^2^-test).

**Results:** The authors analyzed 452 responses of nursing students, and 86.3% of them encountered death of a patient in the course of their studies. In the second-year master's studies, this value reached 99.3%. Among the most frequently mentioned accompanying emotions, students mentioned “reflection on the fragility of life” and “compassion.” Students do not feel sufficiently prepared for the death of a patient.

**Conclusions:** Nursing students encounter the death of a patient very often and very early. Although the curriculum includes content related to the care of a dying patient, students believe that there is still too little of it and that it does not prepare it in a satisfactory manner. Students feel different, often negative emotions related to patient death, and also have various methods of dealing with such a situation. They also feel the need to increase education in this area.

## Introduction

Death is an integral part of human life. Today, death is seen as something that we are afraid of [(1), p. 547–562]. Students very quickly collide with the need to deal with the high stress associated with working with the patient. One of the most difficult moments during studies is the experience of first death of a patient [(2), p. 803–805]. The astonishing amount of literature on this subject is surprising, regarding student voices [(3), p. 634–640; (4), p. 641–647; (5), p. 110; (6), p. 108]. The topic of students' reluctance to work with geriatric patients was also discussed, where it was indicated that it was affected by a high level of fear of death (especially the death of their relatives or patients) [(7), p. 101]. The vast majority of studies concern the experience of patient death by future doctors, although they have to deal with this difficult situation. The literature also deals with the analysis of emotional states accompanying nurses, paramedics and midwives working in the profession [(8), p. 1,442–1,457; (9), p. 594–601; (10), p. 39–49; (11), p. 24–25; (12), p. 100; (13), p. 639–645; (14), p. 402–417]. It was noted that few publications deal with the subject of the patient-death experience and how to deal with it by students of these faculties. In this work, the authors decided to discuss patient death from the perspective of nursing students. Care for a dying patient is an integral part of the work of many nurses. Nursing students deal with this exceedingly early, already during the first years of study. It is especially important to understand what students must face while studying when preparing future nurses for their work. Thanks to this, teachers and mentors can provide appropriate support and properly prepare students.

The research subject was how early and how often nursing students meet a patient death and what emotions and coping methods accompany it. The authors wanted to check the subjective assessment of students' preparation for the death of a patient and their perception of classes conducted in this area.

## Materials and Methods

The research took place in May 2019 using Google Form, which was made available through groups on the Facebook website. The study used the diagnostic survey method, which allowed collecting information about the perspective of nursing students on the perception of aspects related to the death of a patient. The selected tool made it possible to collect data from students of various universities throughout the country, which was very important for the authors. It was dedicated to students of nursing and covered the entire territory of Poland. The researcher did not want to limit himself to nursing students from one academic center to have more objective and varied data. The tool was created based on the analysis of the available literature and the authors' own thoughts [(2), p. 803–805; (8), p. 1,442–1,457; (15), p. 266–272; (16), p. 279–291; (17), p. 448–453; (18), p. 207–228; (19), p. 999–1,007]. A pilot study was carried out to verify the quality of the resulting tool. In this study, 50 people participated.

A total of 467 nursing students participated in this study. Fourteen questionnaires were excluded from the study because of incorrect completion. Four hundred fifty-two responses were qualified for the task (438 female and 14 male participants). Three hundred seven students (67.9%) were on their undergraduate studies, and 145 students (32.1%) were on their graduate studies. Sixty-five respondents were first-year undergraduate students, 121 participants were second-year students, and the same number were third-year students. Eighty-two participants were in the first year of second-degree studies, and 63 in the second year. The above data on the characteristics of the subjects are presented in Table 1.

**Table 1:** Characteristics of study participants (*n* = 452), Poznan, 2019.

**Character**	**Number**	**%**
**Level of education**		
Undergraduate studies in total	307	67.9%
1 year of study	65	14.4%
2 year of study	121	26.8%
3 year of study	121	26.8%
Graduate studies in total	145	32.1%
1 year of study	82	18.1%
2 year of study	63	13.9%
**Sex**		
Female	438	96.9%
Male	14	3.1%

Data collection was carried out with a short questionnaire constructed based on literature analysis and own thoughts. The questionnaire consisted of 14 questions related to basic information about the respondent and his experience related to patient death. There were nine single-choice questions, two multiple-choice questions, two questions based on a Likert scale, and one open question. The questions concerned emotions related to experiencing patient death, ways of coping with the situation, and a sense of preparation for patient-death situation.

The analyzed data were expressed as median, minimum and maximum values, or percentage, as appropriate. Comparison of unpaired groups was performed using the Mann–Whitney *U*-test or the Kruskal–Wallis test with the Dunn *post-hoc* test. The relationship between variables was analyzed with Spearman rank correlation coefficient. Categorical data were analyzed with the χ^2^-test and the Fisher–Freeman–Halton test (by StatXact). All results were considered significant at *p* < 0.05. Statistical analyses were performed with STATISTICA 13.0 (StatSoft Inc.) or StatXact 11.0 (Cytel Inc.).

The conducted study does not bear the characteristics of a medical experiment; therefore, it did not require the approval of the Bioethics Committee in Poland.

## Results

The authors analyzed 452 responses of nursing students regarding their experience of death and the perception of their preparedness to deal with such a situation. The analysis was carried out in three steps: (1) comparison of results in two groups: between undergraduate and graduate students, (2) comparison in three groups: between first-, second-, and third-year students (detailed analysis of undergraduate studies), (3) comparison in two groups: between the first- and second-year graduate studies (detailed analysis of graduate studies). The vast majority of the study participants were women. More than half of the respondents were on their undergraduate studies.

Most of the respondents have already experienced a situation related to patient death. Three hundred ninety participants answered “yes” to this question, which constituted 86.3% of the surveyed population. Sixty-two students (13.7%) said that they have never experienced a death situation during education yet. This result is shown in Table 2. With each year of study, the percentage of participants who met with death increased, until it reached 99.3% in the second year of graduate studies. There is a relationship between the degree of the respondents' studies and their meeting with the death situation (*p* < 0.001). It is weak; the value of the contingency coefficient is 0.25. This means that the vast majority of nursing students experienced a situation connected with patient death in the course of their education, and also in the second year of graduate studies, almost 100% of students experienced this kind of situation.

**Table 2:** Number of students who experienced patients death during nursing education (*n* = 452), Poznan, 2019.

**Have you ever experienced patients death?**		
	**YES**	**NO**
Undergraduate studies	246 (80.1%)	61 (19.9%)
Graduate studies	144 (99.3%)	1 (0.7%)

After the affirmative answer to the question of patient-death experience, the survey automatically redirected to questions related to this experience. It means that this question was answered only by participants who experienced the situation related to patient death during their studies (*n* = 390). In the first year of undergraduate study, most students met with death once or twice. Already in the second year of study, most participants met with death four or more times. This answer is the most frequently chosen answer by students of other years of education. Every year, the disparity between the frequency of choosing “4 or more” and the other answers increases, shown in Table 3. In the first year of education, most of the respondents experienced the death of a patient one to three times, and the median was 2. With education, the value increased, and in the third year of study, most students replied that they had experienced patient death four or more times. There is a significant difference between the graduate and undergraduate students and the number of experienced deaths of patients (*p* < 0.001). Analyzing the responses from first-, second-, and third-year undergraduate students, we get from multiple comparisons test that there is a significant difference between first- and third-year students (*p* < 0.001) and second- and third-year students (*p* < 0.001). The first- and second-year students did not differ in terms of the number of patient-death experiences (*p* = 0.097). There are no significant differences between the first and second year of graduate studies (*p* = 0.059), although the *p* value is slightly higher than 0.05. This means that the further the education stage, the more often students encounter patient death.

**Table 3:** How many times students experienced situation connected with patient's death in each year of studying (*n* = 390), Poznan, 2019.

**Year of education**	**How many times have you experienced situation connected with patient's death?**				
	**1**	**2**	**3**	**4 or more**	**Total**
**Undergraduate studies**					
1	10 (38.5%)	9 (34.6%)	2 (7.7%)	5 (19.2%)	26 (6.7%)
2	25 (24.0%)	21 (20.2%)	19 (18.3%)	39 (37.5%)	104 (26.7%)
3	4 (3.4%)	15 (12.9%)	21 (18.1%)	76 (66.5%)	116 (29.7%)
**Graduate studies**					
1	3 (3.7%)	13 (16.0%)	12 (14.8%)	53 (65.4%)	80 (20.5%)
2	3 (4.8%)	2 (3.2%)	8 (12.7%)	50 (79.4 %)	64 (16.4%)
Total	45 (11.5%)	60 (15.4%)	62 (15.9%)	223 (57.2%)	390

Caring for a patient at the end of life is associated with experiencing many, often difficult emotions and feelings. Another question for students who experienced patient death concerned the emotions associated with this situation. The question was multiple-choice, with no limit to the number of indications. The authors listed the most frequently mentioned emotions in the literature, and the option of adding another answer by themselves was added. The most frequently felt emotion was “reflection on the fragility of life,” then “compassion,” “sadness, depression,” and “helplessness.” Among the answers added by the respondents were “the patient stopped suffering,” “acceptance,” “willingness to see the post-humous toilet.” The answers are shown in Table 4.

**Table 4:** Emotions students felt in the situation connected with patient's death (*n* = 390), Poznan, 2019.

**Emotion**	**Number**
Reflection on the fragility of life	244 (54.0%)
Compassion	220 (48.7%)
Sadness, depression	196 (43.4%)
Helplessness	165 (36.5%)
Calm	85 (18.8%)
Fear	57 (12.6%)
Indifference	51 (11.3%)
A sense of relief	35 (7.7%)
Aversion to the chosen profession	16 (3.5%)
Anger	14 (3.1%)
Other	9 (2.0%)

The authors were also interested in the mechanisms of dealing with patient-death situation. Similarly, to the previous question, the authors listed the most popular ways found in the literature and added the option of adding their answer. Most indications were answered by “I didn't feel the need to cope,” then “I was talking to a close person” and “I was looking for support among my friends.” Twelve participants admitted that they used drugs after this event. Only 10% of respondents had the opportunity or need to talk to a class supervisor. Four participants added their own answers. Among the answers added by the respondents were “I tried not to delve into what happened, death is one of the inseparable elements of work in the hospital,” “I have seen too many patients' deaths to have a problem with it.” These results mean that a significant proportion of students do not feel the need to deal with such a situation. Most of the rest of them seek support from relatives or friends. A summary of the answers to this question is presented in Table 5.

**Table 5:** Methods of coping with the situation connected with patient's death (*n* = 390), Poznan, 2019.

**Method of coping**	**Number**
I did not feel the need to cope	163 (36.1%)
I was talking to a close person	136 (30.1%)
I was looking for support from my friends	90 (19.9%)
I was praying	64 (14.2%)
I was crying	46 (10.2%)
I talked to the class supervisor	45 (10.0%)
I was looking for loneliness	29 (6.4%)
I was looking for entertainment	12 (2.7%)
I used stimulants	12 (2.7%)
Other	4 (0.9%)

The nurse education program assumes preparation for contact with patient-death situation. The authors asked if students felt prepared and compared it with the year of education. Future nurses could rate this on a Likert 5-point scale, where 1 meant “very bad” and 5 meant “very good.” The most common answer was “average,” which choose 36.3% of respondents. Only 10.8% described their preparation as “very good.” There were no statistically significant differences between answers given by undergraduate and graduate students (*p* = 0.949). There was also no statistically significant difference between the first- and second-year graduate students in terms of the level of feeling prepared for the situation related to patient death (*p* = 0.601). There is, however, a statistically significant difference between the responses of participants from the first year and the third year of undergraduate studies (*p* < 0.001). Third-year students rate their preparation for the situation related to patient death higher.

The next question was aimed at examining the need felt by students to introduce more educational classes in the field of preparation to deal with patient-death situation; 81.9% of respondents said that they think the number of classes is insufficient, 8.6% think that the number of classes is sufficient, and 9.5% have no opinion. Table 6 shows the distribution of answers to the question divided into undergraduate and graduate. There was no statistically significant difference between the undergraduate and graduate students (*p* = 0.417). There was no statistically significant difference between first and second year of graduate studies in this question (*p* = 0.265), and there was also no statistically significant difference between the responses of undergraduate students (*p* = 0.363). This means that students, regardless of the level of education, indicated a similar need to increase the number of classes preparing to meet patient-death situation.

**Table 6:** Opinion about missing classes in coping with death during education (*n* = 452), Poznan, 2019.

	**Yes**	**No**	**I have no opinion**
Undergraduate studies	247 (80.5%)	30 (9.8%)	30 (9.8%)
Graduate studies	123 (84.8%)	9 (6.2%)	13 (9.0%)

The last question concerned the extent to which classes during studies prepare students to meet patient-death situation. This question also used a 5-point Likert scale, where 1 meant “very bad” and 5 meant “very good”; 39.4% of students indicated that they assessed these classes at 2, which meant “bad”; 29.6%, “very bad”; and 24.8%, “average.” Undergraduate and graduate students similarly determined the degree to which the classes carried out as part of the study program prepare to meet patient death. There was no statistically significant difference (*p* = 0.736). There was also no statistically significant difference between first and second year of graduate studies in this question (*p* = 0.588). A statistically significant difference was found between the responses of participants from the first and third year of studying (*p* = 0.047, value at the border of statistical significance). Third-year students of the undergraduate studies assessed their preparation to face the situation of patient death higher than students of the first year of education.

There was a significant positive correlation (*p* < 0.001, *r* = 0.29) between the declared number of experiences related to death and the subjective sense of preparation for the situation related to patient death. This correlation exists for both undergraduate (*r* = 0.35) and graduate (*r* = 0.27) students. It means that students gain confidence with the practical experience they acquire. This relationship has been observed among both undergraduate and graduate students.

## Discussion

The conducted research shows how broad and, at the same time, necessary for students this topic is. The authors are aware that the presented results are not exhaustive. The study itself has limitations, primarily because of the chosen tool; not everyone likes and is willing to fill in the online questionnaire (e.g., fear of anonymity). A limitation of this study is also the small number of male participants. The authors also see the need for an in-depth discussion with students, which was not possible during the course of this study. Additionally, representatives of one faculty were examined, and it would be interesting to refer to other groups of medical students.

In modern culture, death is perceived as a two-fold phenomenon—first as something terrifying, shameful, and repulsive, and second as something fascinating. It is also an integral part of the work of medics. Every doctor, nurse, midwife, and physiotherapist will experience patient death in his work. Most people who choose a medical faculty will encounter such a situation during their studies, which is shown by the tests carried out by future doctors [(20), p. 123–133; (5), p. 110; (6), p. 108; (18), p. 207–228]. These results are consistent with our study, which showed that the vast majority of nursing students meet with the death of the patient very early, and almost all participants of master's studies experienced patient death at least once. This result may be because nurses have constant contact with the patient, which, according to Klepacka and Bakalski, is one of the greatest advantages of this profession. They also indicate that patients expect support especially from nurses (21). With each year of education, the number of experiences related to the death of a patient increases. Interestingly, there is a significant difference between the first and third years of undergraduate studies. Graduate students faced such a situation much more often than undergraduate students.

The authors did not find quantitative studies that would clearly indicate the emotions accompanying future doctors in such a situation. However, there are several qualitative studies in which students during interviews, conversations, or writing a diary describe what they felt at the time. There is a significant similarity here. The emotions they exchanged depended on the situation—in the case when patient death was in the hospital, they exchanged grief, sadness, and feeling heartbroken and upset. Completely different emotions accompanied them in the event of a death in the emergency department. Here, mainly shock, confusion, and surprise were mentioned [(5), p. 110]. In this study, respondents most often indicated that they were accompanied by “reflection on the fragility of life.” It is noteworthy that students are usually incredibly young, it is not usual that people of this age are accompanied by such reflections, and it certainly has an impact on their lives and mental health. The next most frequently mentioned emotions were “compassion,” “sadness,” and “helplessness.” These are extremely difficult emotions, especially for young people. What is surprising is that 16 people indicated that after encountering the death of the patient, they felt an aversion to the chosen profession, which means that it was definitely more difficult for them than they could have imagined and predicted while choosing their professional path. The authors of the study from 2017 indicated several factors—patient's age, expectation, or suddenness of death, the first experience, relatable to personal experience, interaction with the patient and his family, and coping mechanism adopted—have emotions that accompany a patient death. Interestingly, the students thought that the reaction of the family was often more difficult for them than the death of the patient [(5), p. 110].

Methods of dealing with patient-death situation of medical and nursing students are also similar. The authors of various studies mention, among others, crying, talking to a loved one, and emotional distance [(18), p. 207–228]. Students also pointed out that meeting with friends and drinking beer helped them cope with the problematic situation [(6), p. 108]. In this study, several people also indicated an escape to intoxicants. This is puzzling because as future medical practitioners, they are aware of the harmful effects of intoxicants, but in difficult situations, they reach for them. In this study, respondents most often indicated “didn't feel the need to cope.” However, the authors wonder if this is due to a real lack of need or a lack of awareness of the need to deal with this situation. Very often students indicated the need to talk to someone close and support among friends. It is interesting that only 10% of respondents had the opportunity or need to talk to a class supervisor. According to the authors, this would be the best option because they are experienced and educated in the field of pedagogy and preparation for the profession. Is this reluctance due to a lack of trust? Are mentors inaccessible? These are the questions that the authors leave open because the available literature cannot answer them.

It was decided to check if nursing students feel prepared for patient-death situation. Third-year students of undergraduate studies felt more prepared for the situation related to patient death compared to first-year undergraduates. However, the analysis of the answers did not show any significant differences between undergraduate and graduate students. These results are puzzling, because when analyzing the data of undergraduate students, one could think that education strengthens the feeling of preparing students for the death of a patient, but the comparison of undergraduate and graduate students does not confirm this hypothesis. However, it can be concluded from the obtained results that people who have more experience (they have encountered patient death more times) feel more prepared for further experiences in this field. This allows the conclusion that practical experience gives a feeling of being better prepared for such experiences. Lack of preparation for patient death was indicated by the respondents in another article, where one of the respondents wrote: “I was just panicking this was just too much it was just awful, they don't prepare you for that” [(17), p. 448–453]. The vast majority of nursing students stated that they lack classes preparing to deal with the death of a patient. This number decreased in the third year of studies but, like the question related to the subjective feeling of being prepared for the death of the patient, increased in master's studies.

Patient death is an experience described by many students as transformative, which teaches a lot and significantly changes the perception of their role in future work. It is often a memory that accompanies the end of life. Nursing students, according to Labrague et al. [(22), p. 279–291], are accompanied by moderate to high levels of stress, which is one of the reasons for patient care and workload. The authors of this study indicate that the key to helping students is to present methods of coping with stress in the education process [(22), p. 279–291]. Moreover, nurses and other healthcare workers are burdened with stressful work, difficult situations, and contact with patients. All of this makes them vulnerable to reduced motivation to work, occupational burnout, and even disease (23, 24). That is why the mentor's support during this time is extremely important, which is also confirmed by other authors [(16), p. 279–291; (17), p. 448–453; (25), p. 760–765; (8), p. 1,442–1,457; (3), p. 634–640; (26), p. 7–17].

The authors also point out that teaching about death, dying, and caring at end of life () takes place over several meetings and is excessively theoretical, and real experiences are left unsaid [(20), p. 123–133]. In 2016, it was proven that simulation could be effectively used to teach about death and dying. The authors emphasized, however, that it must be used properly and responsibly [(27), p. 316–322]. This is confirmed by the creators of the 2018 study on teaching resident doctors to talk about death, where greater effectiveness of simulation-based training was indicated compared to the didactic session. In addition, they showed that even a 30-min conversation can result in increased comfort in working with a dying patient and using the word “|die” [(28), p. 1,221–1,226]. The use of simulation methods is also mentioned by Carmack and Kemery (29). In this review, it was shown that education in the care of dying patients should include didactic teaching, clinical experience, and simulation. They also added that interprofessional education plays an important role [(29), p. 96–100].

The education process is undoubtedly influenced by students' experience that results from their private lives. It is emphasized that knowing and understanding what future medical practitioners experience before starting education are crucial to correctly choose teaching methods to analyze their own experiences and their use in inpatient care [(19), p. 999–1,007]. Proper preparation of mentors and their positive attitude toward students are the key to establishing an educational relationship. According to a study by Bhurtun et al. from 2019, vocational teachers and nursing staff are a vital stress factor because students feel constantly watched and evaluated [(15), p. 266–272] Another study confirming the importance of the teacher in the process of student preparation is the study by Gül et al. from 2019, where they showed that, according to students, a part of teachers avoids patients and families who are considered terminally ill (30). According to Gillian et al., students' experiences are primarily shaped by other people, especially experienced nurses. The authors also point to the need to introduce EOL care education at a very early stage of education. They also point to the vital role of teachers and their attitudes that shape good and bad experiences related to patients' death (31).

Lack of education has a negative impact on the work and attitude of nurses during their professional career. Students feel the need to be more prepared to face the death of the patient. The authors recommend modifying the curriculum to include much more practical classes and exercises, preparing future nurses to experience patient death. Nursing students declare the need for such courses. The authors would also like to emphasize that it is essential to conduct further research on the perception of a patient's death by students of nursing and other medical sciences, their coping methods, and the feeling of being prepared for such an experience.

## Data Availability Statement

The raw data supporting the conclusions of this article will be made available by the authors, without undue reservation.

## Ethics Statement

Ethical review and approval was not required for the study on human participants in accordance with the local legislation and institutional requirements. Written informed consent for participation was not required for this study in accordance with the national legislation and the institutional requirements.

## Author Contributions

MS and KG contributed to conception, design of the study, and created a survey and collected data. MS, PS, and EB were responsible for the research. MR performed the statistical analysis. MS and PS with help from EB wrote sections of the manuscript. All authors contributed to manuscript revision, read, and approved the submitted version.

## Conflict of Interest

The authors declare that the research was conducted in the absence of any commercial or financial relationships that could be construed as a potential conflict of interest.
